# Taurine and Epidermal Growth Factor Belong to the Signature of First-Episode Psychosis

**DOI:** 10.3389/fnins.2016.00331

**Published:** 2016-07-15

**Authors:** Kati Koido, Jürgen Innos, Liina Haring, Mihkel Zilmer, Aigar Ottas, Eero Vasar

**Affiliations:** ^1^Department of Physiology, Institute of Biomedicine and Translational Medicine, University of TartuTartu, Estonia; ^2^Centre of Excellence for Genomics and Translational Medicine, University of TartuTartu, Estonia; ^3^Psychiatry Clinic, Tartu University HospitalTartu, Estonia; ^4^Department of Biochemistry, Institute of Biomedicine and Translational Medicine, University of TartuTartu, Estonia

**Keywords:** taurine, spermine, cytokines, growth factors, epidermal growth factor, NMDA receptors, first-episode psychosis, antipsychotic treatment

## Abstract

This study evaluated the levels of two amino acid derivatives taurine and spermine in first-episode psychosis (FEP) patients and their response to antipsychotic treatment. The levels of taurine and spermine were significantly up-regulated in antipsychotic-naïve FEP patients compared to control subjects (CS). Treatment of FEP patients with antipsychotic drugs significantly reduced the positive symptoms of schizophrenia. This positive effect was accompanied by a significant reduction of taurine and spermine to the levels measured in CS. General linear model was used to establish associations of taurine and spermine with the levels of cytokines and growth factors, measured in our previous experiments using the same study sample. There was a strong association between taurine and epidermal growth factor (EGF). Both biomarkers significantly correlated with the disease symptoms as well as with the effectiveness of antipsychotic treatment. Accordingly one can conclude that taurine and EGF belong to the signature of FEP. Most probably they reflect altered oxidative stress and corrupted function of N-methyl-D-aspartate (NMDA) receptors in FEP.

## Introduction

There is increasing trend to implicate inflammatory processes, elevated oxidative stress and metabolomic alterations to the pathophysiology of psychotic disorders. Recently we demonstrated a link between epidermal growth factor (EGF) and first-episode psychosis (FEP) (Haring et al., 2015). The levels of EGF were significantly upregulated in FEP patients compared to control subjects (CS). Treatment of FEP patients with antipsychotic drugs improved the clinical symptoms and returned EGF to the levels of CS (Haring et al., 2015). Recent pre-clinical research demonstrated that treatment of rodents with EGF induces oxidative stress (Nagano et al., 2015). Tang et al. (2015) showed that EGF receptor signaling upregulates surface expression of the GluN2B-containing N-methyl-D-aspartate (NMDA) receptor and contributes to long-term potentiation in the hippocampus. Moreover, NMDA receptor agonists reverse impaired psychomotor and cognitive functions associated with hippocampal heparin-binding EGF-like growth factor (Hbegf) deficiency in mice (Sasaki et al., 2015). It is noteworthy that the recent opinion of Hardingham and Do (2016) links the development of schizophrenia (SCZ) with alterations in NMDA receptor function and oxidative stress. These factors have been separately linked to SCZ pathogenesis, but evidence now suggests that they are mechanistically interdependent and contribute to a common SCZ-associated pathology (Hardingham and Do, 2016).

A clinical study performed by Samuelsson et al. (2013) established significantly elevated levels of taurine in patients suffering from chronic SCZ. Taurine is a sulfonated beta amino acid derived from methionine and cysteine metabolism, and it is the most abundant free amino acid in humans and plays an important role in several essential biological processes such as bile acid conjugation, maintenance of calcium homeostasis, osmoregulation, and membrane stabilization (Marcinkiewicz and Kontny, 2014). In addition, to functioning as a neurotransmitter and an inhibitory neuromodulator in the central nervous system (Oja and Saransaari, 1996), taurine may also attenuate apoptosis and function as neuroprotectant, antioxidant and immunomodulator (Almarghini et al., 1991; Redmond et al., 1998). Indeed, taurine has been shown to possess potent neuroprotective capacities, including inhibition of glutamate-induced neurotoxicity (Wu et al., 2009). It is apparent that taurine affects the formation of reactive oxidative species (Zhang et al., 2014). Like EGF, taurine targets the GluN2B-containing NMDA receptor subtype (Chan et al., 2015). Besides that there is considerable evidence that some effects of taurine need the presence of spermine. The effect of taurine on ^3^H-MK801 binding, an antagonist of NMDA receptors, was apparent only in the presence of spermine (Chan et al., 2013). Spermine belongs to polyamines and it is formed from the amino acid ornithine. Spermine reduces damage due to reactive oxygen species, permits correct current flow through inwardly rectifying K^+^ channels, controls activity of brain glutamate receptors involved in learning and memory, and affects growth response (Pegg, 2014). Spermine binds to polyamine sites located on GluN2B subunits (Sirrieh et al., 2015). Spermine binding to the extracellular portion of the NMDA receptor results in an increase in apparent glycine affinity (Williams et al., 1994). On the other hand, the gastro-protective effect of taurine also involves interaction with spermine (Motawi et al., 2007).

Taken together, an apparent link exists between EGF, taurine and spermine at least on the level of oxidative stress and regulation of NMDA receptors via GluN2B subunit. Therefore, the main goal of the present study was to measure the serum levels of taurine and spermine in the same samples in which we have previously described alterations in cytokine and growth factors levels (Haring et al., 2015). For that purpose, additional serum samples from the same participants were used. This approach allows us to compare taurine and spermine levels with EGF and other cytokines/growth factors measured in the previous study. First, the levels of taurine and spermine were compared between FEP patients and CS. Second, the effect of 7-months antipsychotic treatment on clinical symptoms, taurine and spermine levels was evaluated in FEP patients. Subsequently, correlations between taurine and spermine, and previously measured cytokines and growth factors (Haring et al., 2015) were analyzed. Finally, to estimate the relationships among variables, we used general linear models (GLM). Based on the study of Haring et al. (2015) we included EGF, IL-1β, IL-2, IL-4, and IL-6 in addition to taurine and spermine into the regression models to establish effects of FEP and antipsychotic treatment on the serum levels of the biomarkers.

## Materials and methods

### Participants

Patients' data for the present investigation were obtained from an ongoing longitudinal Estonian first-episode psychosis study. Thirty-eight FEP patients were recruited from the Psychiatric Clinic of Tartu University Hospital, Estonia. The patients fulfilled the following inclusion criteria: age between 18 and 45; they had experienced a first psychotic episode; the duration of their untreated psychosis had been less than 3 years; they had received no antipsychotic treatment before their first contact with the medical services for psychosis. Patients were excluded from the study if they had psychotic disorders owing to a general medical condition or had substance induced psychosis. FEP diagnoses were based on clinical interviews according to the International Classification of Diseases, Tenth Edition (ICD-10) (World Health Organization, 1992) criteria. Thirty-six FEP patients underwent treatment using antipsychotic medication (two refused) and were included in the follow-up analysis. History of antipsychotic medication was collected from the reviews of patients' medical charts. Patients were treated with various antipsychotic medications according to what was clinically indicated, and treatments changed over the course of the 7 months interval. During the follow-up period, patients received either atypical (*n* = 24), typical (*n* = 1) or mixed (*n* = 11) antipsychotic medication; the mean theoretical chlorpromazine daily dose equivalent (Gardner et al., 2010) range was 80–640. Twenty-eight patients were treated only with antipsychotics, but five patients additionally needed mood stabilizers and 6 patients also received antidepressants or hypnotics.

Thirty-seven mentally healthy subjects participated in the study as CS, and they were recruited by advertising in the same geographical area the FEP patients came from. Both patients and CS were interviewed by experienced psychiatric doctors in order to avoid the inclusion as controls of subjects with mental disorders. Exclusion criteria for the control group also included psychotic disorders among close relatives. The study was approved by the Ethics Review Committee on Human Research of the University of Tartu (Estonia) and carried out in accordance with The Code of Ethics of the World Medical Association. All subjects gave written informed consent in accordance with the Declaration of Helsinki. The sample of this study contains the same participants as our previous study by Haring et al. (2015).

### Procedure

For the FEP patients, general demographic data was recorded at baseline, and fasting blood samples as well as clinical data were collected at two time points: on admission and after the follow-up period (mean duration 7.18 ± 0.73 months). According to our study protocol patients were re-examined after time interval which comprised of the period what was needed for symptom remission and after that followed an additional 6-months period while patients took their medications steadily. Psychotic symptoms severity was identified using the Positive and Negative Syndrome Scale (PANSS) (Kay et al., 1987) positive symptom subscale. Blood samples and demographic data from CS were collected cross-sectionally.

### Blood collection and clinical laboratory measurements

Blood samples of the participants were collected between 09:00 and 11:00 a.m. Blood (5 ml) was sampled using anticoagulant-free tubes and kept for 1 h at 4°C (for platelet activation) before serum was isolated (centrifugation at 2000 rpm for 15 min at 4°C). Serum was kept at −20°C before testing.

### Measurement of metabolites in serum samples

Serum levels of taurine and spermine were determined with the AbsoluteIDQ™ p180 kit (BIOCRATES Life Sciences AG, Innsbruck, Austria) using the flow injection analysis tandem mass spectrometry (FIA-MS/MS) as well as liquid chromatography (LC-MS/MS) technique on a QTRAP 4500 mass-spectrometer (Sciex, USA). All measurements were performed as described in the manufacturer's kit manual. Identification and quantification of the metabolites were achieved using multiple reaction monitoring (MRM) along with internal standards. Calculations of metabolite concentrations were automatically performed by MetIDQ™ software (BIOCRATES Life Sciences AG). The validation of metabolomic and cytokine/growth factor measurements are presented in the Supplementary Material section.

### Statistical analyses

Group differences with regard to demographic measurements were analyzed using the *t*-test, or Chi-square test. The application of Shapiro-Wilk tests indicated that at the group level, values of taurine and spermine were not normally distributed (*p* < 0.05). Mann-Whitney *U*-test was applied to compare the raw data of two independent samples (FEP patients before treatment and CS) and a Wilcoxon signed rank test to compare the two dependent samples (FEP patients pre- and post-treatment condition).

Spearman's rank correlation analysis determined the relationship between serum biomarkers as well as markers and patients' disease symptom scores. GLM was used to demonstrate biomarker levels differences between antipsychotic-naïve FEP patient and CS. To establish treatment effects to serum biomarker levels, GLM between the groups (FEP patients after 7 months treatment with antipsychotics compared to CS) as well as between-subjects (GLM: repeated measures) were utilized. Categorical (disease, gender, smoking status) and continuous (age) covariates were used in the GLM to compare biomarker levels (dependent variables). To study within-subjects differences in biomarker levels, difference between pre- and post-treatment condition was used as independent variable. Because GLM analyses required normally distributed data, biomarker values were log_10_-transformed to approximate normality.

In our previous study (Haring et al., 2015) among the same participants, we found that antipsychotic-naïve FEP patients demonstrated significantly elevated levels of EGF (*Z* = 6.67, *p* < 0.001), IL-6 (*Z* = 3.17, *p* < 0.002), and IL-4 (*Z* = 3.17, *p* < 0.002), and reduced value of IL-1β (*Z* = 3.01, *p* < 0.003) compared to CS. Moreover, in the same study (Haring et al., 2015) we found that 7 months treatment with antipsychotics had significant effect on EGF (*Z* = 5.16, *p* < 0.001), IL-2 (*Z* = 4.78, *p* < 0.001), IL-4 (*Z* = 2.88, *p* = 0.004), and IL-6 (*Z* = 3.34, *p* < 0.001) levels. To establish the effects of the disease and treatment condition on the taurine, spermine as well as abovementioned inflammatory markers and growth factor levels, we simultaneously entered these log_10_-transformed biomarker levels into the GLM.

The statistical analyses were performed using Statistica software (StatSoft Inc., 12th edition) for Windows (StataCorp, 2011). All statistical tests were two-sided, *p* < 0.05 was considered to be statistically significant.

## Results

### General description of the study groups

There were no statistically significant differences between FEP patients and CS in terms of age [*t*_(73)_ = 0.49, *p* = 0.62], and gender [$\chi_{\left(1\right)}^{2}$ = 1.08, *p* = 0.30]. In addition, the differences in tobacco use (8 patients [21.1%] vs. 7 controls [18.9%]) was not statistically significant [$\chi_{\left(1\right)}^{2}$ = 0.05, *p* = 0.82]. As expected, there was a statistically significant 7 months treatment effect on the positive psychotic symptoms (Wilcoxon signed rank test, *Z* = 5.16, *p* < 0.000001), measured by PANSS positive symptom score (median = 29, range 18–43 during the recruitment, and median = 12, range 7–26, at the follow-up period).

### Biomarker level differences among antipsychotic-naïve FEP patients and control subjects

First, the metabolomic measurements demonstrated a robust elevation of taurine (*Z* = 5.79, *p* < 0.000001) in antipsychotic-naïve FEP patients compared to CS (Table 1). The levels of spermine were also significantly increased (*Z* = 3.20, *p* < 0.001) in the patients group (at baseline) compared to CS; however, this elevation was not as prominent as in the case of taurine (Table 1).

**Table 1:** **Comparisons of taurine and spermine levels between the first episode psychosis patients at baseline (FEP_**b**_) and healthy control subjects (HC) as well as FEP patients at baseline (FEP_**b**_) and after 7 months of treatment (FEP_**f**_) with antipsychotics**.

**Biomarkers**	**CS Median (min–max) (pg/mL)**	**FEP_b_ Median (min–max) (pg/mL)**	**FEP_f_ Median (min–max) (pg/mL)**	***Z*-value^a^**	***p*-value^a^**	***Z*-value^b^**	***p*-value^b^**
Taurine	46.30	76.45	46.60	5.79	<0.000001	5.07	<0.000001
	(25.80–78.70)	(32.40–172.00)	(28.20–119.00)				
Spermine	0.23	0.27	0.19	3.20	0.001	2.98	0.003
	(0.16–0.28)	(0.17–0.43)	(0.16–0.27)				

Z-adjusted values

a according to Mann-Whitney U-test (FEP_b_ compared to CS). Z-values

b *according to Wilcoxon Matched Pairs Test (FEP_b_ compared to FEP_f_)*.

Spearman rank correlation analyses revealed significant relationships between taurine and spermine (*r* = 0.68, *p* < 0.000001), taurine and EGF (*r* = 0.63, *p* < 0.000001), as well as less pronounced correlations between taurine and IL-4 (*r* = 0.24, *p* < 0.05), and taurine and IL-1ß (*r* = −0.28, *p* < 0.05) when all the participants were taken into account. In addition, spermine was significantly correlated with EGF (*r* = 0.37, *p* < 0.001).

In the next step, multivariate GLM analysis elucidated that predominantly levels of taurine (*p* < 0.000001) and EGF (*p* < 0.000001) were significantly elevated in antipsychotic-naïve FEP patients compared to CS group. However, patients also demonstrated higher levels of IL-6 (*p* < 0.0003) and IL-4 (*p* < 0.0007) compared to CS. A statistical analysis of the overall difference between the groups is shown in Table 2. Age, gender and smoking status were not significant predictors to differentiate biomarker levels between the groups in our study.

**Table 2:** **Regression coefficients (ß) and significance values of log_**10**_-transformed biomarker levels with disease**.

**Biomarkers**	**ß**	**ß (95% CI)**	***t*-value**	***p*-value**
**AMINO ACID DERIVATIVES**				
Taurine	0.65	0.46−0.83	7.04	<0.000001
Spermine	0.23	−0.01–0.47	1.88	0.06
**GROWTH FACTOR**				
EGF	0.81	0.65−0.96	10.67	<0.000001
**PRO-INFLAMMATORY CYTOKINES**				
IL-1ß	−0.25	−0.49–(−0.01)	−2.05	0.05
IL-2	0.13	−0.12−0.38	1.06	0.29
IL-4	0.41	0.18−0.64	3.58	0.0007
IL-6	0.43	0.20−0.65	3.80	0.0003

*CI, Confidence intervals; EGF, epidermal growth factor; IL-1ß, IL-2, IL-4, IL-6, interleukins*.

When only one predictor (disease) was used to differentiate the combination of taurine, EGF, IL-4, and IL-6 serum levels between antipsychotic-naïve FEP patients and CS, a robust main effect of the disease emerged [*F*_(4, 69)_ = 36.17, *p* < 0.000001, partial eta^2^ = 0.68].

### Antipsychotic treatment effect on biomarker levels among FEP group

After 7-months antipsychotic treatment, FEP patients' taurine and spermine serum concentrations showed a significant decrease (Wilcoxon signed rank test, *Z* = 5.07, *p* < 0.000001 and *Z* = 2.98, *p* = 0.003, respectively) compared with pre-treatment levels (Table 1).

In further analyses we evaluated the simultaneous impact of treatment on taurine and spermine as well as on EGF, IL-1ß, IL-2, IL-4, and IL-6 (growth factor and cytokines which concentrations were from our previous study (Haring et al., 2015) known to be dependent on antipsychotic treatment) levels. Repeated measures GLM was performed to compare the main effect of the 7-months antipsychotic treatment on the concentration of serum biomarkers. A powerful decrease over time was detected in the serum levels of taurine (*p* < 0.000001), EGF (*p* < 0.000001) and IL-2 (*p* < 0.000001). In addition, serum concentrations of IL-4 (*p* < 0.007), IL-6 (*p* < 0.008), and spermine (*p* = 0.02) demonstrated a significant reduction. All effects of the treatment are summarized in Table 3. While a combination of significantly changed biomarker levels were used in the repeated measure GLM, the size of the treatment effect (partial eta^2^) was 0.73, [*F*_(6, 64)_ = 29.34, *p* < 0.000001].

**Table 3:** **Regression coefficients (ß) and significance values of log_**10**_-transformed biomarker levels in first episode patients group before treatment compared to biomarker values measured after 7 months of treatment with antipsychotics**.

**Biomarkers**	**ß**	**ß (95% CI)**	***t*-value**	***p*-value**
**AMINO ACID DERIVATIVES**				
Taurine	0.63	0.44–0.82	6.66	<0.000001
Spermine	0.28	0.05–0.51	2.39	0.02
**GROWTH FACTOR**				
EGF	0.78	0.63–0.93	10.27	<0.000001
**PRO-INFLAMMATORY CYTOKINES**				
IL-1ß	0.11	−0.13–0.35	0.92	0.36
IL-2	0.57	0.37–0.77	5.72	< 0.000001
IL-4	0.32	0.09–0.55	2.78	0.007
IL-6	0.31	0.08–0.54	2.72	0.008

*CI, Confidence intervals; EGF, epidermal growth factor; IL-1ß, IL-2, IL-4, IL-6, interleukins*.

Furthermore, to evaluate the FEP patients' post-treatment status with regard to biomarker levels compared to CS (adjusted for age, gender and smoking status), GLM was performed. As seen from Table 4, the antipsychotic treatment reversed the elevated values of taurine and spermine as well as the levels of EGF, IL-4, and IL-6 to the levels comparable with CS. Interestingly, after 7 months of treatment with antipsychotics, FEP patients demonstrated significantly lower levels of IL-1ß and IL-2 compared to CS.

**Table 4:** **Regression coefficients (ß) and significance values of log_**10**_-transformed biomarker levels in first episode patients group after 7 months of treatment with antipsychotics compared to control subjects**.

**Biomarkers**	**ß**	**ß (95% CI)**	***t*-value**	***p*-value**
**AMINO ACID DERIVATIVES**				
Taurine	0.09	−0.17–0.34	0.69	0.50
Spermine	−0.04	−0.29–0.21	−0.31	0.76
**GROWTH FACTOR**				
EGF	−0.05	−0.30–0.20	−0.40	0.69
**PRO-INFLAMMATORY CYTOKINES**				
IL-1ß	−0.33	−0.57–(−0.09)	−2.76	0.008
IL-2	−0.49	−0.71–(−0.28)	−4.55	0.00003
IL-4	0.10	−0.15–0.35	0.81	0.42
IL-6	0.22	−0.03–0.47	1.79	0.08

*CI, Confidence intervals; EGF, epidermal growth factor; IL-1ß, IL-2, IL-4, IL-6, interleukins*.

## Discussion

The present study demonstrated that the serum levels of taurine were significantly increased in antipsychotic-naïve FEP patients. This change was comparable to that established for EGF in our previous study (Haring et al., 2015). By contrast, the alteration of another biomarker spermine, mediating some effects of taurine (Motawi et al., 2007; Chan et al., 2013), was less pronounced in these FEP patients. These findings are in accordance with the research performed by Bjerkenstedt et al. (1985) and Samuelsson et al. (2013) demonstrating a significant increase of taurine in patients suffering from SCZ. Bjerkenstedt et al. (1985) performed the study in antipsychotic-free patients, whereas the study of Samuelsson et al. (2013) was conducted in patients under treatment with antipsychotic drugs. However, neither of these studies engaged antipsychotic-naïve FEP patients. Therefore, the present study underlines that elevation of taurine is already present in the early stage of SCZ manifestation. It is remarkable that taurine displayed a significant positive correlation with EGF in FEP patients. The relation of spermine to EGF was markedly weaker compared to its interaction with taurine. GLM supported a strong association of taurine and EGF with the disease in antipsychotic-naïve FEP patients group.

Existing evidence shows that EGF and taurine are implicated in the regulation of oxidative stress (Chen et al., 2012; Nagano et al., 2015) and activity of NMDA receptors (Chan et al., 2015; Tang et al., 2015). It is apparent that both EGF and taurine affect the formation of reactive oxidative species (Cha et al., 2000; Zhang et al., 2014). On the other hand, the redox status has been shown to play a role in the regulation of NMDA receptors (Hashimoto, 2014). Moreover, recent studies demonstrate that both EGF and taurine influence the GluN2B subunit of NMDA receptors (Chan et al., 2015; Tang et al., 2015). Therefore, one could speculate that the elevated levels of EGF and taurine in antipsychotic-naïve FEP patients may reflect the corrupted function of NMDA receptors. It is important to underline that both biomarkers penetrate the blood-brain barrier (Pan and Kastin, 1999; Salimäki et al., 2003) and, therefore, peripherally circulating molecules may potentially interact with NMDA receptors in the brain. It is well known that the blocking of NMDA receptors with antagonists like ketamine induces psychotic symptoms in healthy volunteers and causes exacerbation of psychotic symptoms in patients suffering from SCZ (Lahti et al., 2001). Therefore, one could suggest that the elevation of taurine and EGF may reflect suppressed function of NMDA receptors in FEP patients. Moreover, the gene determining the GluN2A subunit of NMDA receptors and the gene determining serine racemase, an enzyme that generates NMDA receptor co-agonist D-serine, were determined to be among the risk genes for SCZ (Ripke et al., 2014).

Treatment with antipsychotic drugs ameliorated clinical symptoms in FEP patients. Besides that the treatment reduced the upregulated values of taurine and spermine to the levels established for CS. This positive change is similar as determined for several inflammatory and growth factors [EGF, IL-2, IL-4, IL-6, vascular endothelial growth factor (VEGF) and interferon-gamma (IFN-γ)] in our previous study (Haring et al., 2015). The decrease of EGF and taurine was in line with the decline of PANSS positive symptom score. The reduction of spermine was also in significant correlation with taurine and EGF. It is important to underline that antipsychotic treatment reduced clinical symptoms, but also reversed the levels of EGF, taurine and spermine to the level of CS. Taking into account the role of taurine and EGF in the regulation of oxidative stress and activity of NMDA receptors one could suggest that antipsychotic treatment restored the function of NMDA receptors impaired by FEP. Application of GLM also underlines that the reduction of EGF and taurine levels reflects the positive effect of antipsychotic drugs.

To the best of our knowledge there are no available studies examining the covariation of taurine, spermine, EGF and inflammatory biomarkers in antipsychotic-naïve FEP patients compared to CS, and establishing impact of long-term antipsychotic drug therapy on simultaneous alterations of above-mentioned biomarkers. Our data emphasizes the idea that the peripheral biomarkers can be used to detect mental illness. The discovery of novel diagnostic, prognostic or therapeutically useful biomarkers is of immense importance for the understanding of the underlying physiopathology of SCZ. Unfortunately, a particular biomarker with ideal specificity and sensitivity is difficult to find, and one potential solution is to use the combinational power of different biomarkers. It is believed that by integrating potential biomarkers into drug development and clinical use, a more personalized approach to patients could ultimately be adopted.

Some limitations also need to be acknowledged. First, we collected blood samples from CS at one point in time and did not control their biomarker levels after the same time interval as was done for FEP patients. Second, during the re-evaluation, we recruited patients who were receiving antipsychotic treatment, and we demonstrated antipsychotic treatment effects on the levels of taurine, spermine, EGF and inflammatory markers in FEP patients group. While different drugs have different affinities for mono-aminergic receptors, they may also have a different impact on biomarkers' levels. However, because of our relative small sample size and the naturalistic study design we could not establish direct effects of any specific active substance on the measured biomarkers. Additional results from larger samples of individuals with FEP are required to confirm and expand our results in terms of specific antipsychotic drug, dosage and treatment duration. Furthermore, FEP studies suggest that antipsychotics may not be a major contributor to peripheral biomarker levels (Di Nicola et al., 2013) and changes in serum levels of biomarkers found in patients with psychotic disorder could be part of the disease pathology rather than a direct change resulting from treatment (Pillai et al., 2015).

In conclusion, we established a significant co-upregulation of taurine and EGF in antipsychotic-naïve FEP patients. This change probably reflects a relationship between FEP and impaired function of NMDA receptors. Antipsychotic treatment was associated with the reduction of positive symptoms and with the return of levels of EGF and taurine to that of established for CS. Therefore, one could suggest that long-term antipsychotic treatment tends to modify the function of NMDA receptors.

## Author contributions

KK was responsible for handling the study samples and revising the manuscript, JI was responsible for statistical analysis and editing the manuscript, LH as professional psychiatrist took care of the clinical studies and collecting of blood samples, MZ and AO were responsible for metabolomics measurements, and EV designed the experiment, analyzed the results and wrote the manuscript. All the authors of the manuscript have approved the final version of the manuscript.

## Funding

This research was supported by the grants from the Estonian Research Foundation (IUT 20-41, IUT 20-42, IUT 20-45). This research was supported by the European Union through the European Regional Development Fund (Project No. 2014-2020.4.01.15-0012).

### Conflict of interest statement

The authors declare that the research was conducted in the absence of any commercial or financial relationships that could be construed as a potential conflict of interest.

## References

[B1] Almarghini K. Remy A. Tappaz M. (1991). Immunocytochemistry of the taurine biosynthesis enzyme, cysteine sulfinate decarboxylase, in the cerebellum: evidence for a glial licalization. Neuroscience 43, 111–119. 10.1016/0306-4522(91)90421-J 1922763

[B2] Bjerkenstedt L. Edman G. Hagenfeldt L. Sedvall G. Wiesel F. A. (1985). Plasma amino acids in relation to cerebrospinal fluid monoamine metabolites in schizophrenic patients and healthy controls. Br. J. Psychiatry 147, 276–282. 10.1192/bjp.147.3.276 2415198

[B3] Cha Y. K. Kim Y. H. Ahn Y. H. Koh J. Y. (2000). Epidermal growth factor induces oxidative neuronal injury in cortical culture. J. Neurochem. 75, 298–303. 10.1046/j.1471-4159.2000.0750298.x 10854274

[B4] Chan C. Y. Singh I. Magnuson H. Zohaib M. Bakshi K. P. Le François B. (2015). Taurine targets the GluN2b-containing NMDA receptor subtype, in Taurine 9 [Internet], eds Marcinkiewicz J. Schaffer S. W. (Cham: Springer International Publishing), 531–44. 10.1007/978-3-319-15126-7_43 25833525

[B5] Chan C. Y. Sun H. S. Shah S. M. Agovic M. S. Ho I. Friedman E. (2013). Direct Interaction of taurine with the NMDA glutamate receptor subtype via multiple mechanisms, in Taurine 8 [Internet], eds El Idrissi A. L. L'Amoreaux W. J. (New York, NY: Springer), 45–52. 10.1007/978-1-4614-6130-2_4 23392923

[B6] Chen G. Nan C. Tian J. Jean-Charles P. Li Y. Weissbach H.. (2012). Protective effects of taurine against oxidative stress in the heart of MsrA knockout mice. J. Cell. Biochem. 113, 3559–3566. 10.1002/jcb.24233 22740506

[B7] Di Nicola M. Cattaneo A. Hepgul N. Di Forti M. Aitchison K. J. Janiri L.. (2013). Serum and gene expression profile of cytokines in first-episode psychosis. Brain Behav. Immun. 31, 90–95. 10.1016/j.bbi.2012.06.010 22749891 PMC3675681

[B8] Gardner D. M. Murphy A. L. O'Donnell H. Centorrino F. Baldessarini R. J. (2010). International consensus study of antipsychotic dosing. Am. J. Psychiatry 167, 686–693. 10.1176/appi.ajp.2009.09060802 20360319

[B9] Hardingham G. E. Do K. Q. (2016). Linking early-life NMDAR hypofunction and oxidative stress in schizophrenia pathogenesis. Nat. Rev. Neurosci. 17, 125–134. 10.1038/nrn.2015.19 26763624

[B10] Haring L. Koido K. Vasar V. Leping V. Zilmer K. Zilmer M.. (2015). Antipsychotic treatment reduces psychotic symptoms and markers of low-grade inflammation in first episode psychosis patients, but increases their body mass index. Schizophr. Res. 169, 22–29. 10.1016/j.schres.2015.08.027 26364730

[B11] Hashimoto K. (2014). Targeting of NMDA receptors in new treatments for schizophrenia. Expert Opin. Ther. Targets. 18, 1049–1063. 10.1517/14728222.2014.934225 24965576

[B12] Kay S. R. Flszbein A. Opfer L. A. (1987). The Positive and Negative Syndrome Scale (PANSS) for Schizophrenia. Schizophr. Bull. 13, 261–276. 10.1093/schbul/13.2.261 3616518

[B13] Lahti A. C. Weiler M. A. Tamara Michaelidis B. A. Parwani A. Tamminga C. A. (2001). Effects of ketamine in normal and schizophrenic volunteers. Neuropsychopharmacology 25, 455–467. 10.1016/S0893-133X(01)00243-3 11557159

[B14] Marcinkiewicz J. Kontny E. (2014). Taurine and inflammatory diseases. Amino Acids. 46, 7–20. 10.1007/s00726-012-1361-4 22810731 PMC3894431

[B15] Motawi T. K. Abd Elgawad H. M. Shahin N. N. (2007). Modulation of indomethacin-induced gastric injury by spermine and taurine in rats. J. Biochem. Mol. Toxicol. 21, 280–288. 10.1002/jbt.20194 17912696

[B16] Nagano T. Mizuno M. Morita K. Nawa H. (2015). Pathological implications of oxidative stress in patients and animal models with schizophrenia: the role of epidermal growth factor receptor signaling. Curr. Top Behav. Neurosci. 10.1007/7854_2015_399. [Epub ahead of print]. 26475158

[B17] Oja S. S. Saransaari P. (1996). Taurine as osmoregulator and neuromodulator in the brain. Metab. Brain Dis. 11, 153–164. 10.1007/BF02069502 8776717

[B18] World Health Organization (1992). The ICD-10 Classification of Mental and Behavioural Disorders: Clinical Descriptions and Diagnostic Guidelines. Geneva: World Health Organization.

[B19] Pan W. Kastin A. J. (1999). Entry of EGF into brain is rapid and saturable. Peptides 20, 1091–1098. 10.1016/S0196-9781(99)00094-7 10499427

[B20] Pegg A. E. (2014). The function of spermine: function of Spermine. IUBMB Life 66, 8–18. 10.1002/iub.1237 24395705

[B21] Pillai A. Howell K. R. Ahmed A. O. Weinberg D. Allen K. M. Bruggemann J.. (2015). Association of serum VEGF levels with prefrontal cortex volume in schizophrenia. Mol. Psychiatry 21, 686–692. 10.1038/mp.2015.96 26169975

[B22] Redmond H. P. Stapleton P. P. Neary P. Bouchier-Hayes D. (1998). Immunonutrition: the role of taurine. Nutrition 14, 599–604. 10.1016/S0899-9007(98)00097-5 9684263

[B23] Ripke S. Neale B. M. Corvin A. Walters J. T. R. Farh K. H. Holmans P. A.. (2014). Biological insights from 108 schizophrenia-associated genetic loci. Nature 511, 421–427. 10.1038/nature13595 25056061 PMC4112379

[B24] Salimäki J. Scriba G. Piepponen T. P. Rautolahti N. Ahtee L. (2003). The effects of systemically administered taurine and N-pivaloyltaurine on striatal extracellular dopamine and taurine in freely moving rats. Naunyn Schmiedebergs Arch. Pharmacol. 368, 134–141. 10.1007/s00210-003-0776-6 12898127

[B25] Samuelsson M. Skogh E. Lundberg K. Vrethem M. Öllinger K. (2013). Taurine and glutathione in plasma and cerebrospinal fluid in olanzapine treated patients with schizophrenia. Psychiatry Res. 210, 819–824. 10.1016/j.psychres.2013.09.014 24113127

[B26] Sasaki K. Omotuyi O. I. Ueda M. Shinohara K. Ueda H. (2015). NMDA receptor agonists reverse impaired psychomotor and cognitive functions associated with hippocampal Hbegf-deficiency in mice. Mol. Brain 8:83. 10.1186/s13041-015-0176-0 26637193 PMC4670538

[B27] Sirrieh R. E. MacLean D. M. Jayaraman V. (2015). Subtype-dependent *N* -Methyl-d-aspartate Receptor Amino-terminal domain conformations and modulation by Spermine. J. Biol. Chem. 290, 12812–12820. 10.1074/jbc.M115.649723 25829490 PMC4432297

[B28] StataCorp (2011). Stata Statistical Software: Release 12. College Station, TX: StataCorp, LP.

[B29] Tang Y. Ye M. Du Y. Qiu X. Lv X. Yang W.. (2015). EGFR signaling upregulates surface expression of the GluN2B-containing NMDA receptor and contributes to long-term potentiation in the hippocampus. Neuroscience 304, 109–121. 10.1016/j.neuroscience.2015.07.021 26204818

[B30] Williams K. Zappia A. M. Pritchett D. B. Shen Y. M. Molinoff P. B. (1994). Sensitivity of the N-methyl-D-aspartate receptor to polyamines is controlled by NR2 subunits. Mol. Pharmacol. 45, 803–809. 8190097

[B31] Wu J. Y. Wu H. Jin Y. Wei J. Sha D. Prentice H. (2009). Mechanism of neuroprotective function of taurine, in Taurine 7 [Internet], eds Azuma J. Schaffer S. W. Ito T. (New York, NY: Springer), 169–79.

[B32] Zhang Z. Liu D. Yi B. Liao Z. Tang L. Yin D.. (2014). Taurine supplementation reduces oxidative stress and protects the liver in an iron-overload murine model. Mol. Med. Rep. 10, 2255–2262. 10.3892/mmr.2014.2544 25201602 PMC4199407

