# Supplementary material for: Taurine and Epidermal Growth Factor Belong to the Signature of First-Episode Psychosis
Source: Front Neurosci. 2016 Jul 15;10:331. doi: 10.3389/fnins.2016.00331 (PMC4945631; doi:10.3389/fnins.2016.00331)
Supplement: Supplementary file 1 [file DataSheet1.DOCX]

**Supplementary material**

**Taurine and Epidermal Growth Factor Belong to the Signature of First-Episode Psychosis**

Kati Koido, Jürgen Innos, Liina Haring, Mihkel Zilmer, Aigar Ottas, Eero Vasar*

*Correspondence - Eero Vasar, E-mail: [eero.vasar@ut.ee](mailto:eero.vasar@ut.ee)

1. **Measurement of spermine and taurine**

The Absolute*IDQ*® p180 Kit was validated for human EDTA plasma. Both EDTA and heparin are suitable anticoagulants. Due to its biological similarity, human serum can also be used without impairing the analytical performance. With the Absolute*IDQ* p180 Kit, amino acids and biogenic amines are quantified by stable isotopes in the LC-MS/MS mode. Therefore, in contrast to the p150 Kit and in contrast to analytes that are detected in flow injection (FIA) mode, seven different concentration levels of (external) standard mixes are provided as lyophilized material with the p180 Kit. The dissolved standards are used as calibration standards in order to perform the quantification in the MS-specific software. There are three different quality control levels (QCs) to be positioned on the plate as described in the user manual. The controls represent lyophilized human plasma samples (anticoagulant: EDTA) with respectively low, medium and high (spiked) concentration levels. Results are displayed in the MetVal module of Met*IDQ* and are also shown in the results table of the MetStat module. In MetVal, some of the quality controls often appear with an invalid status. In most cases, this happens due to single outliers. Due to the high number of analytes that are measured with the Kit, unspecific analytical variations may appear leading to single outliers.

**Table S1. Taurine and spermine**

|  | Analyte | | Quality Type Liquid Chromatography | | Evaluated Quantification | | |
| --- | --- | --- | --- | --- | --- | --- | --- |
|  | MetIDQ Short Name | Biochemical Name |  |  | LOD (μM) | LLOQ (μM) | ULOQ (μM) |
| 1 | Spermine | Spermine |  | X | 0.5 | 0.25 | 20 |
| 2 | Taurine | Taurine | X |  | 0.5 | 2.5 | 200 |

LOD – limits of detection

LLOQ – lower limit of quantification

ULOQ – upper limit of quantification

**Calibration, precision and accuracy**

To determine the validation parameters of the LC-MS/MS part, calibration curves were generated using a standard mix with all amino acids and biogenic amines that are covered by the LC part at 7 different concentration levels.

Linear or quadratic regressions were performed using data of six repetitive preparations by plotting the mean concentrations of the replicates against the theoretical amount. R^2^ values were calculated, whereby taurine and spermine exhibited values of R^2^ ≥ 0.990. Table 2 shows the regression parameters for taurine and spermine, respectively. The values are based on the AB SCIEX 4000 QTRAP®, but are similar for all other MS platforms.

**Table S2. Regression and precision of spermine and taurine**

| *Analyte* | *Regression* | *Standard / Calibrator levels (CV)* | | | | | | |
| --- | --- | --- | --- | --- | --- | --- | --- | --- |
| *MetIDQ Short Name* | *R2* | *Cal1* | *Cal2* | *Cal3* | *Cal4* | *Cal5* | *Cal6* | *Cal7* |
| Spermine | 0.9904 | 1% | 1% | 3% | 3% | 6% | 8% | 8% |
| Taurine | 0.9989 | 4% | 7% | 4% | 4% | 5% | 7% | 7% |

The accuracy of the measurements were determined for each calibration level, as summarized in Table 3 for taurine and serine, respectively. All analyses exhibited valid accuracies in the range of 88 – 107 %. The values are based on the AB SCIEX 4000 QTRAP®, but are similar for all other MS platforms.

**Table S3. Accuracy of spermine and taurine**

| *Analyte* | *Standard / Calibrator levels (Accuracy)* | | | | | | |
| --- | --- | --- | --- | --- | --- | --- | --- |
| *MetIDQ Short Name* | *Cal1* | *Cal2* | *Cal3* | *Cal4* | *Cal5* | *Cal6* | *Cal7* |
| Spermine | 92% | 88% | 105% | 107% | 102% | 89% | 99% |
| Taurine | 97% | 100% | 105% | 100% | 98% | 99% | 102% |

**Reproducibility**

The reproducibility of the Kit was determined by assaying three spiking levels of plasma (low, medium, high) using the available standards with n=6 replicates on 3 different MS instruments. For spermine and taurine, the inter-day precision and accuracy was determined at three different levels of spiked human pool plasma. For taurine and spermine, plasma level “low” is diluted plasma (1:2 with PBS, 4% HSA), level “medium” is plasma spiked with Cal2, and level “high” is plasma spiked with Cal3. The validation results are summarized in Table 4. The values are based on the AB SCIEX 4000 QTRAP®, but are similar for all other MS platforms.

**Table S4. Inter-day precision and accuracy of spermine and taurine in matrix (human pool plasma)**

| *Analyte* | *Plasma low* | | *Plasma medium* | | *Plasma high* | |
| --- | --- | --- | --- | --- | --- | --- |
| *MetIDQ Short Name* | *CV* | *Accuracy* | *CV* | *Accuracy* | *CV* | *Accuracy* |
| Spermine | 12% | 73% | 20% | 92% | 26% | 139% |
| Taurine | 5% | 101% | 11% | 103% | 12% | 105% |

CV – coefficient variation

*Note:* In general, the determination of inter-day accuracies of endogenous analytes in plasma is a complicated task since handling, storage conditions and temperature of the matrix may strongly influence the results. For instance, analyte-converting enzymes or analyte-binding proteins could be more or less active. Therefore, strong deviations (from the validation criteria) at non-physiological high concentration levels can be detected for some of the analytes, and these are not taken into account to describe the overall validation status.

1. **Measurement of cytokines and growth factors**

High-sensitive biochip array technology (Cytokine & Growth array for Evidence Investigator™, Randox Biochip, RANDOX Laboratories Ltd, Crumlin, U.K.) was used to perform simultaneous quantitative detection of multiple analytes from a single serum sample (Haring et al., 2015). The following cytokines and growth factors were measured according to the manufacturer’s protocol: TNF-α, IFN-γ, IL-1α, IL-1β, IL-2, IL-4, IL-6, IL-8, IL-10, MCP-1, VEGF and EGF. Results are expressed as picograms per milliliter. The reproducibility of the assay for individual cytokines was determined using the quality control method provided with the kit (Haring et al., 2015).

Intra-assay precision was determined by assessing 20 replicates of each of three levels of sample and is shown in Table S5. Inter-assay precision was determined by assessing 2 replicates of each of three levels of sample in 10 separate assays and is shown in Table S6. Sensitivity of cytokine assay is shown in Table S7.

**Table S5. Intra-assay precision of high sensitive cytokine array**

| Analyte | Level 1 | | Level 2 | | Level 3 | |
| --- | --- | --- | --- | --- | --- | --- |
|  | Concentration pg/ml | CV % | Concentration pg/ml | CV % | Concentration pg/ml | CV % |
| IL-1α | 2.7 | 10.9 | 45.4 | 9.7 | 169.7 | 11.4 |
| IL-1ß | 5.1 | 9.3 | 63.9 | 8.7 | 233.4 | 7.4 |
| IL-2 | 16.7 | 7.8 | 84.1 | 5.8 | 255.8 | 6.9 |
| IL-4 | 13.1 | 9.6 | 69.9 | 9.5 | 232.2 | 8.4 |
| IL-6 | 1.4 | 11.9 | 10.5 | 9.8 | 26.7 | 7.8 |
| IL-8 | 11.6 | 9.4 | 123.0 | 9.4 | 629.0 | 7.0 |
| IL-10 | 8.2 | 6.8 | 93.9 | 5.6 | 286.0 | 6.1 |
| IFN-γ | 7.9 | 10.1 | 145.7 | 7.4 | 620.1 | 7.7 |
| TNF-α | 9.4 | 7.1 | 111.8 | 7.2 | 486.1 | 12.7 |
| MCP-1 | 27.6 | 12.2 | 136.3 | 8.9 | 320.8 | 5.8 |
| VEGF | 17.3 | 10.4 | 146.4 | 10.8 | 456.0 | 7.3 |
| EGF | 13.0 | 9.2 | 36.7 | 9.5 | 198.7 | 9.6 |

CV: coefficient of variation.

IL-1α, IL-1β, IL-2, IL-4, IL-6, IL-8, IL-10: interleukins; IFN-γ: interferon-γ;

TNF-α: tumor-necrosis factor-α; MCP-1: monocyte chemo-attractant protein-1;

VEGF: vascular endothelial growth factor; EGF: epidermal growth factor

**Table S6**. **Inter-assay precision of high sensitive cytokine array**

| Analyte | Level 1 | | Level 2 | | Level 3 | |
| --- | --- | --- | --- | --- | --- | --- |
|  | Concentration  pg/ml | CV % | Concentration  pg/ml | CV % | Concentration  pg/ml | CV  % |
| IL-1α | 2.7 | 15.5 | 51.1 | 8.9 | 174.9 | 13.4 |
| IL-1ß | 4.2 | 9.9 | 66.7 | 11.8 | 213.7 | 8.7 |
| IL-2 | 16.4 | 7.8 | 85.2 | 6.5 | 258.9 | 8.2 |
| IL-4 | 12.5 | 11.6 | 71.4 | 8.6 | 206.9 | 8.8 |
| IL-6 | 1.5 | 8.4 | 10.3 | 7.4 | 23.7 | 8.4 |
| IL-8 | 11.3 | 11.1 | 132.6 | 9.9 | 560.8 | 9.2 |
| IL-10 | 8.2 | 6.7 | 96.8 | 6.5 | 239.4 | 7.5 |
| IFN-γ | 8.1 | 11.4 | 135.4 | 6.4 | 542.1 | 7.6 |
| TNF-α | 9.9 | 8.6 | 116.5 | 6.7 | 442.1 | 7.0 |
| MCP-1 | 27.9 | 12.8 | 139.8 | 10.4 | 272.4 | 7.2 |
| VEGF | 17.4 | 12.0 | 135.2 | 7.2 | 395.7 | 10.7 |
| EGF | 13.1 | 11.7 | 38.9 | 8.5 | 181.2 | 9.6 |

CV: coefficient of variation.

IL-1α, IL-1β, IL-2, IL-4, IL-6, IL-8, IL-10: interleukins; IFN-γ: interferon-γ;

TNF-α: tumor-necrosis factor-α; MCP-1: monocyte chemo-attractant protein-1;

VEGF: vascular endothelial growth factor; EGF: epidermal growth factor.

**Table S7**. **Sensitivity of cytokine assay**

| Analyte | Calibration range (pg/ml) | Sensitivity (pg/ml) |
| --- | --- | --- |
| IL-1α | 0 – 225 | 0.19 |
| IL-1ß | 0 – 1125 | 0.26 |
| IL-2 | 0 – 1200 | 0.90 |
| IL-4 | 0 – 1500 | 2.12 |
| IL-6 | 0 – 400 | 0.12 |
| IL-8 | 0 – 1450 | 0.38 |
| IL-10 | 0 – 450 | 0.37 |
| IFN-γ | 0 – 500 | 0.44 |
| TNF-α | 0 – 600 | 0.59 |
| MCP-1 | 0 – 500 | 0.66 |
| VEGF | 0 – 1000 | 1.53 |
| EGF | 0 – 450 | 1.04 |

IL-1α, IL-1β, IL-2, IL-4, IL-6, IL-8, IL-10: interleukins; IFN-γ: interferon-γ; TNF-α: tumor-necrosis factor-α; MCP-1: monocyte chemo-attractant protein-1; VEGF: vascular endothelial growth factor; EGF: epidermal growth factor.

**Reference**

Haring L, Koido K, Vasar V, Leping V, Zilmer K, Zilmer M, et al. Antipsychotic treatment reduces psychotic symptoms and markers of low-grade inflammation in first episode psychosis patients, but increases their body mass index. Schizophr Res. 2015 Dec;169(1–3):22–9.
